# Sarcoidal tattoo granuloma after COVID-19 vaccine

**DOI:** 10.1016/j.jdcr.2023.06.022

**Published:** 2023-06-28

**Authors:** Courtney N. Haller, Alicia Schnebelen, Simi D. Cadmus

**Affiliations:** a Division of Dermatology, Department of Internal Medicine, Dell Medical School, the University of Texas at Austin, Austin, Texas; b Dermpath Diagnostics, Arlington, Texas

**Keywords:** COVID-19, tattoo sarcoid, vaccination

## Introduction

Sarcoidosis is an inflammatory multisystemic disease characterized by noncaseating granulomas that can affect multiple organ systems. The thoracic cavity is most commonly affected with pulmonary tissue and surrounding lymph node inflammation. The next most commonly affected system is the skin, seen in up to 35% of patients.^1^ Not everyone with skin involvement will have systemic sarcoidosis. In those who present first with cutaneous signs and symptoms, reports on whether these patients also have systemic manifestations of sarcoidosis range from 30% to 85% in the literature.^2^ Sarcoid granulomata can occur as an idiopathic process or can be provoked in skin with prior trauma, such as in scars or tattoos.^3^^,^^4^

There are few reports of cutaneous sarcoidosis appearing after COVID-19 or vaccination or both. We present the case of a 39-year-old woman with cutaneous sarcoid nodules affecting tattoos shortly after COVID-19 vaccination. Our case is rare because the nodules were present in only some tattoos and developed within various tattoo ink colors.

## Case report

**Fig 1:**
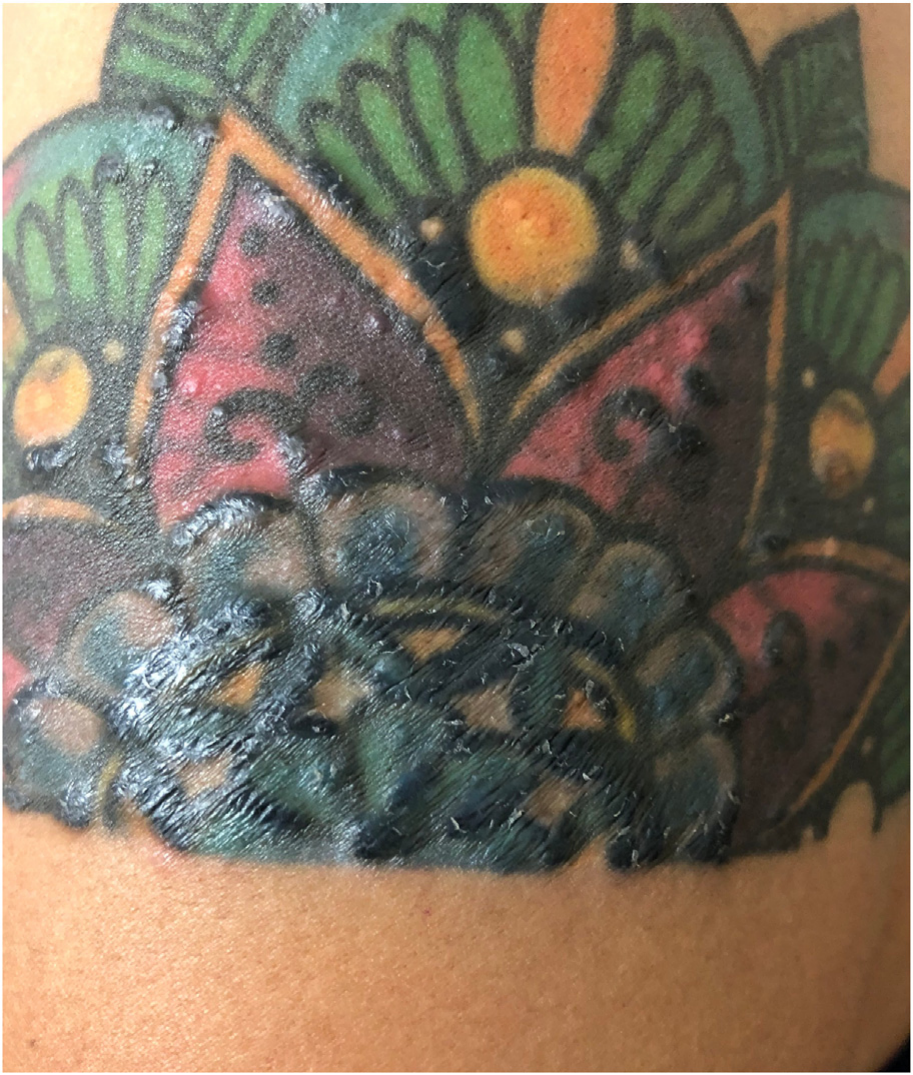
Sarcoidal firm papules present within the multicolored tattoo on the upper portion of the left arm.

**Fig 2:**
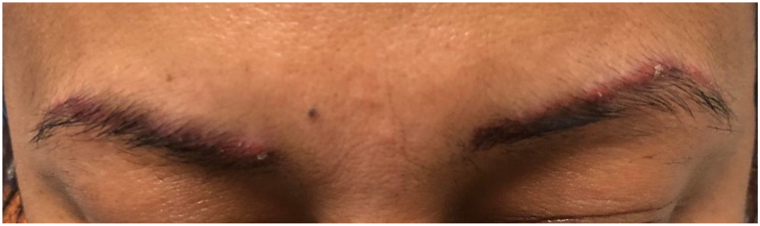
Eyebrows with firm papules within red/dark brown tattoo pigment.

A 39-year-old Hispanic woman presented to the dermatology clinic for a pruritic rash within 3 of her 4 tattoos. Affected areas included a multicolored ink tattoo (black, green, red, and blue) on the upper portion of her left arm present for 6 years and dark brown/red-inked tattooed eyebrows present for 8 years. She had an additional black-inked tattoo on the lower portion of her back (present for 14 years) that did not develop a rash. She noticed rashes on her eyebrows and the upper portion of her left arm appeared on her tattoos approximately 1 month after receiving a second dose of the Moderna COVID-19 vaccine. She had received her first dose of the same vaccine brand 28 days beforehand. She denied a rash at the injection site. Other than a history of mild psoriasis on the elbows and vitamin D deficiency, she was otherwise healthy and denied illness or prior history of SARS-CoV-2 infection prior to the rash onset. Further review of systems was negative. Examination showed several papules and nodules located within the tattoos on the upper portion of her left arm (Fig 1) and eyebrows (Fig 2). The tattoo on the lower portion of her back did not have any nodules.

**Fig 3:**
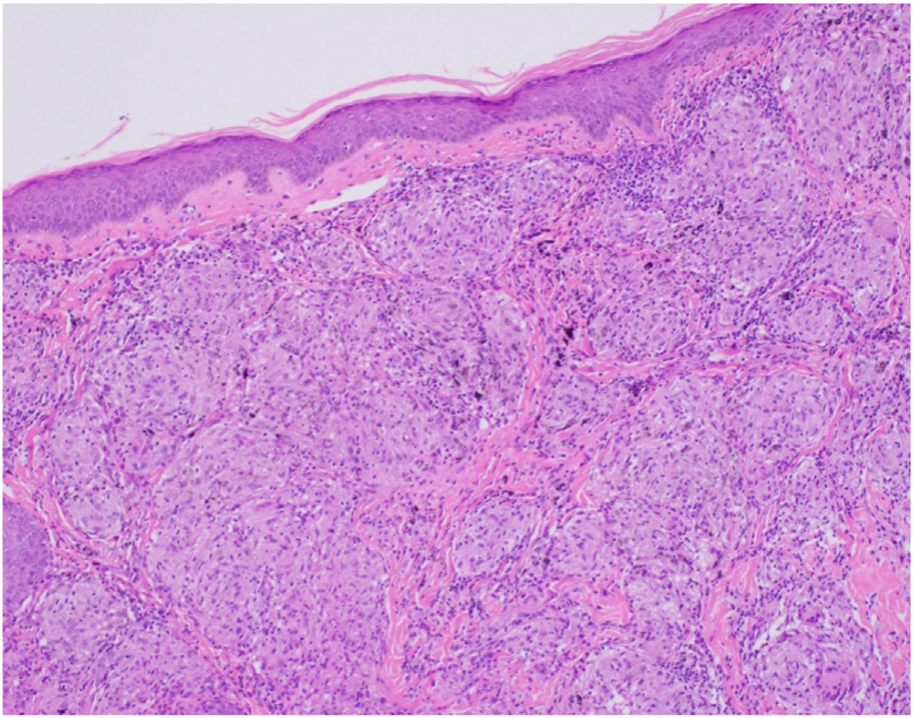
Sarcoidal dermal granulomata without caseation are present on tissue pathology with hematoxylin and eosin.

A punch biopsy of the tattoo on the upper portion of her left arm revealed an unremarkable epidermis, with dermal nodular aggregates of giant histiocytes with a mild associated lymphocytic infiltrate without caseation (Fig 3). Grocott methenamine silver, periodic acid–Schiff, and Fite’s acid-fast stains did not reveal infectious organisms. These findings were consistent with sarcoid-like cutaneous reaction to tattoo pigment.

Complete blood cell count, comprehensive metabolic panel, angiotensin-1-converting enzyme levels, and thyroid-stimulating hormone levels were within normal limits. Vitamin D serum level was slightly low; however, the patient had a known history of vitamin D deficiency. Urinalysis was negative for proteinuria. Antinuclear antibody was 1:80. Infectious workup, including QuantiFERON gold, rapid plasma reagin, and HIV serum testing were negative. Chest radiograph was unremarkable. Further workup for systemic sarcoidosis was incomplete because the patient was lost to follow-up.

The patient was initially treated with topical hydrocortisone cream 2.5% for the eyebrows and topical clobetasol cream 0.05% with minimal improvement. Therefore, an oral prednisone taper of approximately 1 mg/kg for 3 weeks was initiated, leading to significant improvement in pruritus and flattening of both the eyebrow and arm tattoo nodules.

## Discussion

Cutaneous sarcoidosis can manifest in areas that were previously traumatized, such as in tattoos. This reaction can be triggered by an immunologic phenomenon, although there are few reports in the literature. An associated potential trigger in our case for the patient’s tattoo sarcoidal granulomata was COVID-19 vaccination. According to the Naranjo scale for adverse drug reactions, our patient’s probability of the vaccine triggering the granulomatous rash is “possible.”^5^ Although it is difficult to make a direct correlation, our observed finding is important to consider. Our case is rare because nodules were present in some but not all tattoos and did not discriminate against multiple ink colors. Colored tattoos are the most likely culprit for skin reactions, as compared with black, of which red is most notorious.^6^ These colored pigments may contain several metals and other potentially toxic compounds that are difficult to identify and may trigger the immune system.

Due to the recent development of the COVID-19 vaccines, there is a paucity of evidence regarding their cutaneous adverse effects in the literature. A review of published trial data from vaccine manufacturers showed that apart from the most common side effect of local injection site reaction with <0.2% of those who received vaccinations with cases of associated contact dermatitis, eczema, urticaria, acneiform eruptions, etc, with a few rashes listed as unspecified, and no specific mention of tattoo granuloma.^7^ Further, 2 observational studies looked at real-world cutaneous reactions after COVID-19 vaccination with 50 and 803 cutaneous reactions after COVID-19 vaccinations reported, respectively. The most commonly reported cutaneous adverse effects in these studies included injection site reaction, urticaria or angioedema, and eruption of papules and plaques with spongiotic dermatoses.^8^^,^^9^ From these studies, only one case of a tattoo sarcoidal granuloma after the Moderna COVID-19 vaccine was reported, although there are no further details because it was a large registry-based study.^9^ Further, there are only a few cases reports in the literature of patients developing cutaneous sarcoidal granulomas after COVID-19 or vaccination, in either normal or previously scarred, noninjured, or tattooed skin.^10^, ^11^, ^12^, ^13^ Interestingly, there is a case report of COVID-19 vaccine induced red-dye tattooed lip reaction with cheilitis although a biopsy was not performed.^14^

As with other chronic diseases, patients with sarcoidosis are thought to be at increased risk of worse outcomes when infected with COVID-19 compared with healthy individuals. However, there is a paucity of data to understand the outcomes of the vaccination on patients with sarcoidosis.^15^ Further, the vaccine itself may trigger the onset of sarcoidosis in an otherwise healthy individual. However, this does not preclude individuals from completing the vaccination series, especially given potentially worse outcomes of COVID-19. More research is needed because there are sparse cases of cutaneous tattoo sarcoidal granuloma after COVID-19 vaccination in the literature.

## Conflicts of interest

None disclosed.
